# The randomised thoracoscopic talc poudrage+indwelling pleural catheters versus thoracoscopic talc poudrage only in malignant pleural effusion trial (TACTIC): study protocol for a randomised controlled trial

**DOI:** 10.1136/bmjresp-2023-001682

**Published:** 2023-05-30

**Authors:** Alexandra Dipper, Anand Sundaralingam, Emma Hedley, Emma Tucker, Paul White, Rahul Bhatnagar, Andrew Moore, Melissa Dobson, Ramon Luengo-Fernandez, Janet Mills, Sandra Sowden, John E Harvey, Lee Dobson, Robert F Miller, Mohammed Munavvar, Najib Rahman, Nick Maskell

**Affiliations:** 1 Academic Respiratory Unit, University of Bristol, Bristol, UK; 2 Oxford Respiratory Trials Unit, University of Oxford, Oxford, UK; 3 School of Data and Mathematics, University of the West of England, Bristol, UK; 4 Respiratory Medicine, North Bristol NHS Trust, Bristol, UK; 5 Translational Health Sciences Musculoskeletal Research Unit, University of Bristol Medical School, Bristol, UK; 6 Respiratory Medicine, Lancashire Teaching Hospitals NHS Foundation Trust, Preston, UK; 7 Respiratory Medicine, Royal Devon and Exeter NHS Foundation Trust Hospital, Exeter, UK; 8 Institute for Global Health, University College London, London, UK; 9 School of Medicine, University of Central Lancashire, Preston, UK; 10 Oxford NIHR Biomedical Research Centre, Oxford, UK

**Keywords:** Lung Cancer, Pleural Disease

## Abstract

**Introduction:**

Malignant pleural effusion (MPE) is common, with 50 000 new cases per year in the UK. MPE causes disabling breathlessness and indicates advanced disease with a poor prognosis. Treatment approaches focus on symptom relief and optimising quality of life (QoL). Patients who newly present with MPE commonly require procedural intervention for both diagnosis and therapeutic benefit.

Thoracoscopic pleural biopsies are highly sensitive in diagnosing pleural malignancy. Talc poudrage may be delivered at thoracoscopy (TTP) to prevent effusion recurrence by effecting pleurodesis. Indwelling pleural catheters (IPCs) offer an alternative strategy for fluid control, enabling outpatient management and are often used as ‘rescue’ therapy following pleurodesis failure or in cases of ‘trapped lung’. It is unknown whether combining a TTP with IPC insertion will improve patient symptoms or reduce time spent in the hospital.

The randomised thoracoscopic talc poudrage + indwelling pleural catheters versus thoracoscopic talc poudrage only in malignant pleural effusion trial (TACTIC) is the first randomised controlled trial (RCT) to examine the benefit of a combined TTP and IPC procedure, evaluating cost-effectiveness and patient-centred outcomes such as symptoms and QoL. The study remains in active recruitment and has the potential to radically transform the pathway for all patients presenting with MPE.

**Methods and analysis:**

TACTIC is an unblinded, multicentre, RCT comparing the combination of TTP with an IPC to TTP alone. Co-primary outcomes are time spent in the hospital and mean breathlessness score over 4 weeks postprocedure. The study will recruit 124 patients and aims to define the optimal pathway for patients presenting with symptomatic MPE.

**Ethics and dissemination:**

TACTIC is sponsored by North Bristol NHS Trust and has been granted ethical approval by the London-Brent Research Ethics Committee (REC ref: 21/LO/0495). Publication of results in a peer-reviewed journal and conference presentations are anticipated.

**Trial registration:**

ISRCTN 11058680.

**WHAT IS ALREADY KNOWN ON THIS TOPIC**

- Definitive pleural intervention is advised to achieve symptom control in malignant pleural effusion (MPE). This may take the form of talc pleurodesis, which typically necessitates an inpatient admission of up to 7 days, or by inserting an indwelling pleural catheter (IPC). Two observational series have examined thoracoscopic talc pleurodesis (TTP) and IPC as a combined procedure, suggesting high pleurodesis rates, outpatient management and no safety concerns.

**WHAT THIS STUDY ADDS**

- No randomised controlled trial (RCT) has yet compared a combined TTP and IPC procedure early in the patient pathway against standard care (TTP), using validated patient-reported outcome measures. TACTIC is the first trial to robustly challenge the hypothesis that TTP with IPC insertion offers effective pleurodesis at the time of diagnostic biopsy with complete outpatient management, evaluating outcomes that are highly important to patients such as symptoms and quality of life.

**HOW THIS STUDY MIGHT AFFECT RESEARCH, PRACTICE OR POLICY**

- The TACTIC trial is a multicentre RCT with the potential to radically affect how patients with MPE are treated. By streamlining both diagnostic and therapeutic pathways into a single ambulatory procedure, a positive trial outcome is likely to result in this approach being widely adopted by healthcare systems.

## Introduction

Malignant pleural effusion (MPE) occurs when cancer metastasises to the pleura (most commonly from lung cancer in men and breast cancer in women) or in mesothelioma, when the pleura itself is the primary site of malignancy.^1 2^ It is common, with 50 000 new cases per year in the UK^3^ and incidence is likely to rise as global cancer rates increase.^4^ MPE causes disabling breathlessness and indicates advanced disease, with a median survival of 3–12 months.^1^ Treatment focuses on symptom control and optimising quality of life (QoL).

For most patients who newly present with a potential MPE, there is both a diagnostic and therapeutic need. An initial thoracocentesis to obtain pleural fluid (PF) for analysis and to relieve breathlessness is the first step in the standard care pathway, with cytology diagnostic in 46% (95% CI 42% to 58%).^5^ When MPE is suspected but PF is non-diagnostic, pleural biopsies are required and obtained via thoracoscopy or image guided techniques. Diagnostic sensitivity of biopsies obtained at medical thoracoscopy is >93%.^6^

The majority of patients will experience fluid re-accumulation following initial drainage, with associated symptom recurrence. Definitive intervention to provide long-term symptom relief is therefore advised.^1 4^ This may take the form of pleurodesis, a procedure where the lung is adhered to the chest wall using talc as a chemical sclerosant to prevent fluid recurrence. Talc may be delivered at thoracoscopy (‘talc poudrage’) or as talc ‘slurry’ by chest tube following fluid drainage, with no difference in pleurodesis efficacy between these methods (36/161 (22%) pleurodesis failures vs 38/159 (24%), respectively).^7^ While thoracoscopy and talc poudrage (TTP) allows the operator to offer pleurodesis in the same sitting as tissue biopsy (combining diagnosis and treatment in a single procedure) both TTP and talc slurry pleurodesis typically confer an inpatient hospital stay of up to 7 days.^8 9^ In addition to the resource pressures of modern-day healthcare systems, elective hospital admission in the context of poor estimated survival may be unacceptable to the patient for a variety of reasons.

Indwelling pleural catheters (IPCs) are an alternative method for definitive fluid management, offering comparable breathlessness control to talc pleurodesis and improved QoL.^10^ IPCs are tunnelled catheters, which are inserted as a day-case procedure and can remain in situ long term, allowing PF drainage to be conducted at home. They can be used where pleurodesis has failed and are also an option for patients who have ‘trapped lung’ (where the lung is unable to expand when fluid is drained), which occurs in 15% of patients with MPE.^1 8^

### Rationale for the study

**Figure 1:**
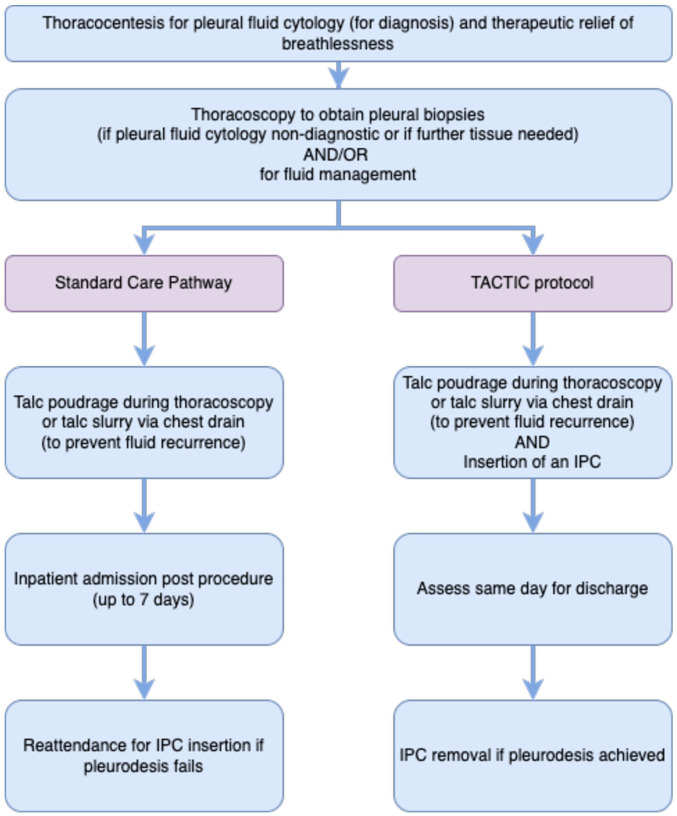
Comparison of standard care patient pathway and TACTIC protocol. IPC, indwelling pleural catheter.

The current standard care patient pathway and protocol for the randomised thoracoscopic talc poudrage + indwelling pleural catheters versus thoracoscopic talc poudrage only in malignant pleural effusion trial (TACTIC) are highlighted in figure 1.

A procedure combining medical thoracoscopy, talc poudrage and IPC insertion (TTP and IPC) may overcome the usual requirements for an inpatient admission following talc pleurodesis. A combined procedure can mitigate the risk of pleurodesis failure following TTP (including as a result of ‘trapped lung’), as patients can continue to drain their effusion using the IPC and effectively control their symptoms without the need for further invasive interventions. TTP with IPC insertion may therefore also provide more effective symptom control, better QoL and cost savings to the healthcare system.

Two observational series have examined TTP and IPC insertion as a combined procedure, suggesting high pleurodesis rates (92%) at 1 month, median time to IPC removal of <14 days, no significant safety concerns and the ability to deliver the procedure in the outpatient setting.^11 12^ However, these studies were uncontrolled and did not assess QoL or patient reported symptoms. No randomised controlled trial (RCT) has yet compared a combined TTP and IPC procedure early in the patient pathway against standard care (TTP) using patient-reported outcome measures validated for use in the MPE population^13^ and qualitative methods to evaluate and characterise the impact of this intervention on patients and carers.

## Methods and analysis

TACTIC is a non-commercial, multicentre RCT based in UK secondary and tertiary care hospitals. Patients will be recruited from 10 centres with established thoracoscopy and IPC services. A list of participating sites can be found in online supplemental appendix E.

10.1136/bmjresp-2023-001682.supp1 Supplementary data

### Population

We aim to recruit 124 participants with symptomatic MPE, who will be undergoing TTP for effusion control. Both patients in whom MPE is suspected, but not yet confirmed, as well as those with a confirmed diagnosis of MPE are eligible. Participants will be identified by clinicians working in pleural services in the selected centres only, from any part of the pleural service (inpatient, outpatient and procedure lists). They will be screened using the inclusion and exclusion criteria:

Inclusion criteria:

1. Symptomatic pleural effusion and any of the following:
  - Thoracoscopically confirmed evidence of malignant pleural disease (visible cancer at thoracoscopy) which requires talc poudrage as part of routine clinical care.
  - An established diagnosis of MPE (via biopsy or cytology) which requires drainage and pleurodesis as per standard care, where the patient and operator decide on a thoracoscopic treatment.
  - Symptomatic effusion requiring drainage and pleurodesis in the context of established metastatic disease.
2. Able to consent to trial inclusion.

Exclusion criteria:

1. Technically unable to undergo TTP (eg, gross respiratory failure, uncorrectable clotting, unable to tolerate position, significant suspicion of underlying trapped lung or poor performance status).
2. Visual impairment (precluding use of symptom measurement instruments).
3. Previous talc pleurodesis within the last 3 months on ipsilateral side.
4. No means of phone contact.
5. Age <18 years.
6. Females who are pregnant or lactating.
7. Unable to consent to trial inclusion.

Radiological confirmation of a pleural effusion with a chest X-ray (CXR) or thoracic ultrasound (USS) within 2 weeks of the screening consultation is required. In all cases, USS should be performed before randomisation to ensure there is sufficient fluid to conduct a thoracoscopy.

### Consent and randomisation

Participation in the trial will be discussed with the patient at the appropriate consultation, which will form part of their normal care pathway. Written informed consent will be received. Patients with suspected malignancy who are undergoing thoracoscopy for both diagnosis and symptom control will be advised that if pleural appearances during the procedure are not consistent with frank cancerous change, they will not be randomised and will not therefore continue with trial participation. Participants will be informed that they have a right to withdraw from the trial at any time, without having to give a reason and that this will not affect their future care.

Patients will be randomised on a 1:1 basis to receive the trial procedure (TTP+IPC) or standard care (TTP alone) using a web-based randomisation system. Patients with an established diagnosis of MPE may be randomised on the day of their procedure, or up to 48 hours in advance. Where a diagnosis of MPE is not established but findings at thoracoscopy are consistent with malignant disease, randomisation will occur during the procedure. Only patients with findings at thoracoscopy consistent with frank malignant change will be randomised. Patients will not be entered into the trial if there is diagnostic doubt.

Blinding for this study is not feasible given that the TTP+IPC arm will be discharged with an IPC in place.

### Intervention

**Figure 2:**
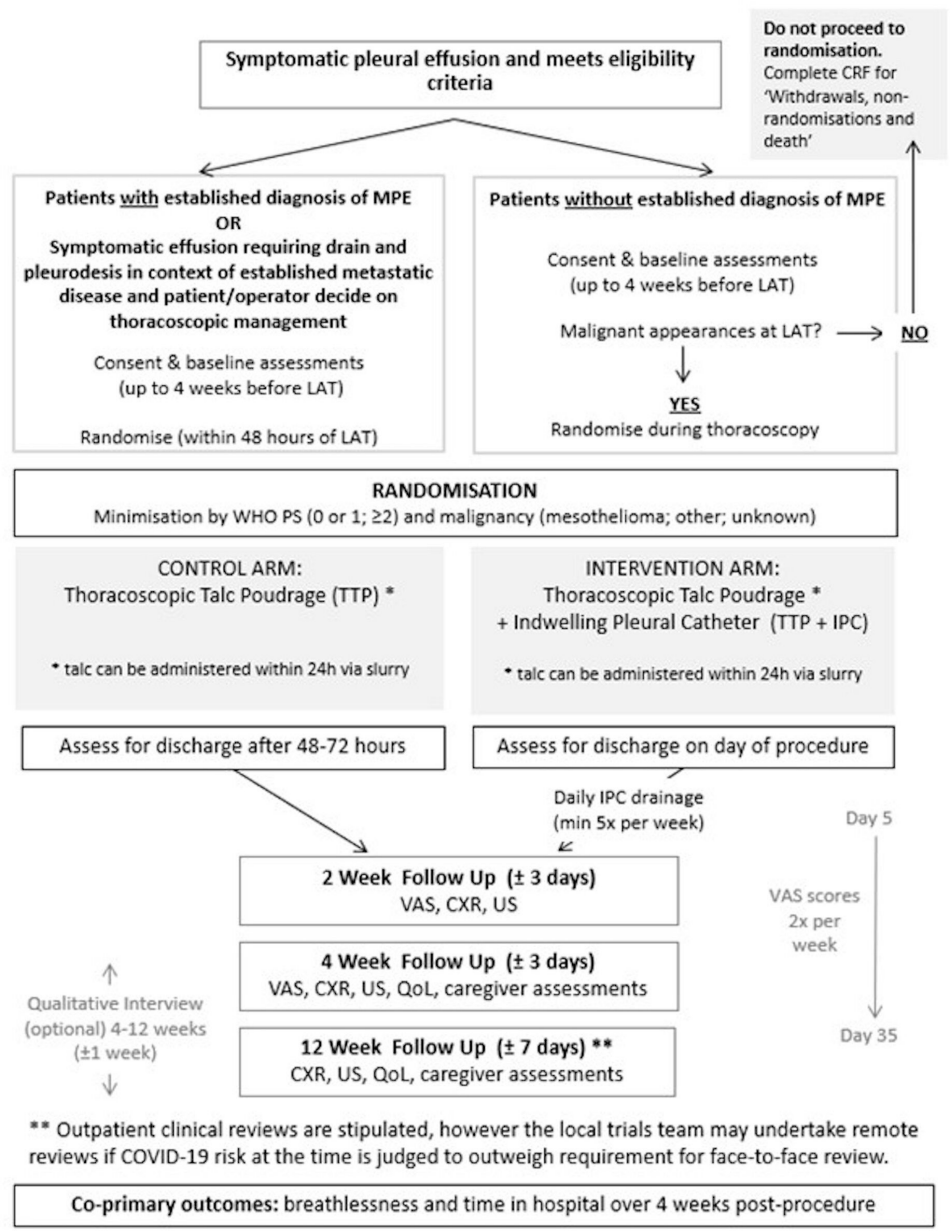
Trial flow chart. CRF, case report form; CXR, chest X-ray; LAT, local anaesthetic thoracsocopy; MPE, malignant pleural effusion; QoL, quality of life; US, ultrasound; VAS, visual analogue scale; WHO PS, World Health Organisation Performance Status.

Trial interventions are summarised by figure 2 and in the schedule of study procedures in online supplemental appendix A.

All patients will undergo a TTP. Those randomised to the intervention arm will have an IPC inserted in addition. TACTIC will provide sites with IPCs (Rocket medical) but sites may choose to use alternative equivalent devices. If talc poudrage at time of thoracoscopy is not possible, for example, due to patient discomfort, it is possible for patients to proceed within the trial if talc slurry is administered within 24 hours of the thoracoscopy. These patients should continue to receive their allocated treatment (IPC or standard care). If patients in either arm do not receive either a talc poudrage or talc slurry (within 24 hours), this would be considered a treatment failure. If an IPC is not sited for patients in the intervention arm, this would also be considered a treatment failure. In cases of treatment failure, clinical care should continue as per usual practice, a protocol deviation should be completed with the appropriate case report form (CRF) and patients should continue with trial follow-up and assessments.

Medical thoracoscopy will be performed according to local practice, but consistent with the British Thoracic Society guideline.^6^ Specific to TACTIC participation, sites are advised to insert the IPC prior to talc poudrage (if the patient is allocated to the intervention arm) to avoid potential difficulty placing the IPC if the patient experiences discomfort post-poudrage. It is advised that the IPC should be inserted through a separate entry to the thoracoscopy port and all patients should have a large bore drain (16–24 Fr) inserted via the port site. Talc poudrage or slurry should be administered with 3–4 g sterile graded talc.

TTP participants will be admitted to hospital postprocedure as per standard care and discharged at the discretion of the treating clinician. TTP+IPC participants will be assessed for discharge on the same day as the intervention but may be admitted if clinically required. For patients who remain an inpatient the trial team will record the day on which suggested discharge criteria (outlined in a trial specific procedure) were met and the day on which discharge occurred.

For intervention arm participants, IPC drainages will be performed by a trained healthcare professional, a minimum of 5 times per week until the first clinic review at 14 days. Participants will be advised to report to the local trial team if PF drains <50 mL on three consecutive drainages. Following this, a clinical review within 7 days will be arranged to consider repeat imaging and IPC removal, which should take place any time up to 14 days after this point. Any patient who has their IPC removed will continue to undergo planned follow-up for the full 12 weeks.

All participants will be given patient diaries to capture visual analogue scale (VAS) scores for breathlessness and chest pain, recorded two times a week over 4 weeks post procedure. Diaries will also capture interaction with healthcare providers. Participants randomised to the intervention arm will record IPC drainage volumes. An extract of the patient diary is included in online supplemental appendix B.

All concomitant medications (other than other intrapleural pleurodesis agents) are permitted in this trial. Use of medications which are thought to reduce pleurodesis success (such as steroids) are permitted but will be recorded on CRFs. There are no trial restrictions, and participants can take part in other (non-pleural interventional) studies if the other trial protocols permit this. Participants in the non-IPC arm are permitted use of IPCs during the post pleurodesis phase if PF becomes recurrent, and this will be recorded on the CRFs. All other forms of pleurodesis (including via the IPC if present) are not permitted during the first month of follow-up.

### Follow-up visits

Participants will be followed up for 12 weeks after the trial procedure, or until death. Trial visits will take place at 2 weeks±3 days, 4 weeks±3 days and 12 weeks±7 days post procedure. At each visit, participants will undergo a standard clinical assessment, including CXR and thoracic USS. QoL questionnaires (EuroQol 5-Dimension 5-Level(EQ5D-5L) and European Organisation for Research and Treatment of Cancer Quality of Life of cancer patients (EORTC QLQc30) will be completed at 4 weeks and 12 weeks. Adverse events (AEs) will also be recorded. Loss to follow-up will be minimised by diligent liaison with the patient, their oncology team and general practitioner. Any loss to follow-up will be recorded on the withdrawal CRF. The reason for withdrawal (if known) will be recorded.

### Carer questionnaires

As an optional aspect of the trial, primary care givers will be asked to complete questionnaires to capture the impact of informal care giving. These will include the Short Form -36 (SF-36); a 5-item scale capturing physical, financial, emotional, social and overall impacts of care burden and the Work Productivity and Activity Impairment as adapted for caregiving (WPAI (CG)) which will ask about productivity losses. Questionnaires will be answered at baseline, 4 weeks and 12 weeks following the patient’s trial procedure.

### Qualitative interview

Patients and their carers (spouse, family or friend) will be invited to participate in a qualitative interview to explore the patient experience of the trial procedures, to characterise the impact of treatment on patient and carer, management of the condition, well-being and QoL. Interviews will be conducted between 4 and 12 weeks (+1 week) following the trial intervention. Patients and carers may be approached about participating in a qualitative interview at any time during the trial period.

### Study outcomes

Our primary research question is:

‘In symptomatic patients with suspected or established MPE, does a combined TTP and IPC result in a reduction in time in hospital and an improvement in dyspnoea, when compared with TTP alone?’

Two co-primary outcomes were chosen to reflect patient priorities in the treatment of MPE, based on patient and public involvement (PPI) feedback. Specifically, these co-primary outcomes will measure total length of stay in hospital (including re-admissions) and average breathlessness scores over 4 weeks postprocedure.

Time spent in hospital will be calculated in whole days, with an overnight admission or continuous period in hospital of >12 hours constituting 1 day. Mean breathlessness scores will be assessed using a 100 mm VAS for dyspnoea. This is validated in patients with MPE undergoing pleural aspiration and has a defined minimal clinically significant difference. VAS dyspnoea scores will be collected at baseline, day 1 postprocedure, day 5 postprocedure and then two times a week for 4 weeks.

Our secondary outcomes are:

1. Effect of TTP+IPC on chest pain over 4 weeks, measured two times a week using a 100 mm VAS score for pain.
2. Total length of stay in hospital over 12 weeks post procedure.
3. Total length of stay in hospital over 4 weeks post procedure, once time spent in hospital for non-medical reasons is discounted. Assessed as total number of days until medically appropriate for discharge according to criteria specified in a trial specific procedure.
4. Pleurodesis success, measured at 4 and 12 weeks post procedure. Defined as absence of a pleural effusion of at least moderate size radiologically (greater than or equal to one-third of hemithorax on CXR or >2 rib spaces and 4 cm depth on ultrasound) AND no further requirement for pleural procedures for effusion control, OR for TTP+IPC participants an IPC output of <50 mL on three consecutive drainages.
5. Healthcare contacts over 12 weeks post procedure, captured using questionnaires and patient diaries.
6. QoL measured using the EQ5D-5L and EORTC QLQc30 questionnaires at baseline, 4 and 12 weeks post procedure.
7. Cost effectiveness of the interventions.
8. Caregiver burden captured using SF-36, WPAI (CG) and 5 item scale questionnaires to capture informal caregiving, measured at baseline, 4 and 12 weeks.
9. Patient and carer experiences of TTP+IPC compared with TTP alone, captured by semistructured qualitative interviews.

### Data management

Data will be securely stored in line with the principles of Good Clinical Practice (GCP) standards and General Data Protection Regulation. Trial Specific Procedures and the data monitoring plan will be followed to ensure quality control and primary outcome data. This trial will use REDCap which is a dedicated and validated clinical trial database designed for data capture.

VAS scores will be measured at sites by two assessors and each assessors measurement uploaded to the REDCap database. If there is a discrepancy of ≥3 mm between the two values, assessors will be prompted to check measurements.

### Trial infrastructure

The Trial Management Group (TMG) is responsible for the day-to-day management of the trial and is responsible for all aspects of the project. As the trial is unblinded the TMG will also review AEs at monthly meetings. In the absence of a Data Monitoring Committee, the Oxford Respiratory Trials Unit (ORTU) Safety Oversight Group will advise the Trial Steering Committee (TSC) on issues related to safety data resulting from the trial.

The TSC provides overall supervision of the study to ensure that it is being conducted in accordance with the protocol, relevant regulations and the principles of Good Clinical Practice. The TSC includes independent members as well as researchers working on the trial. The sponsor will be represented at TSC meetings.

### Safety reporting

Both study arms entail procedures which are standard of care and have associated recognised risks. The population of patients involved in TACTIC is one in which a high number of AEs are expected. Many of these will not be causally related to the investigational intervention, but rather a direct consequence of the patient’s underlying malignancy (which is an entry criteria).

The trial intervention is the combination of an IPC with TTP, therefore only AEs related to this IPC require expedited reporting. For participants in the standard care arm, serious adverse events (SAEs) will be reportable if related to the trial intervention and not a recognised procedural risk. To support assessment of relatedness and causality, a clearly defined list of anticipated events is included in the protocol.

### Sample size and statistical analysis

The statistical analysis plan is included in online supplemental appendix C.

Separate sample size calculations were undertaken for both co-primary outcomes. Using data on hospital length of stay from previous literature,^14^ to detect a difference of 2 days over 4 weeks, with 90% power, a 5% significance level and 5% loss to follow-up, we would require 124 participants. Previous RCTs have demonstrated that mean VAS dyspnoea scores in patients post treatment over 6 weeks is 25 mm (SD 26 mm) when treated with either talc or IPC.^14^ The minimal clinically importance difference (MCID) for VAS dyspnoea is 19 mm.^13^ To detect a smaller difference than the MCID of 16 mm, with 90% power, a 5% significance level and 5% loss to follow-up, we would require a total of 116 participants. The planned sample size of 124 participants will therefore be over-powered for this outcome.

The co-primary outcomes will be initially compared between groups (raw analysis) on an intention to treat basis^15^ using independent t-tests for normally distributed data and non-parametric methods (or correction if skewed) for non-normally distributed data. Adjusted analysis using regression for baseline imbalance and the minimisation factors will then be conducted to increase statistical power and certainty.^16^ Pre-hoc subgroup analyses include referral pathway (cytology confirmation or visual confirmation) and the presence of trapped lung. Missing data will be minimised but major remaining missing data (eg, primary outcome) will be dealt with using imputation and sensitivity analyses.^15^

A full statistical analysis plan will be written and signed off by the TSC before the end of trial recruitment and prior to the analysis of any primary or secondary outcome.

Healthcare costs will be compared between treatment groups over 12 weeks from procedure and valued using NHS reference costs. Cost-effectiveness will be measured using an economic evaluation adherent to guidelines for good practice. A within-trial cost utility analysis will explore incremental cost per Quality Adjusted Life Year gained of TTP+IPC compared with TTP alone.

Qualitative data will be analysed thematically. Up to 24 patient and carer dyads will be interviewed (12 from each treatment arm), however the final sample size will depend on when data saturation is reached, such that further interviews provide no new data.^17^

### Patient and public involvement

ORTU and the Bristol Academic Respiratory Unit have established PPI mechanisms specific to respiratory and pleural disease. Through these contacts, a patient with personal experience of MPE assessed the funding application and commented positively on the research idea, design and lay summary.

This study was designed after discussion with the ORTU patient group, a local patient support group for Mesothelioma and Lung Cancer (the Sunflower Club) and after a survey of 30 patients with MPE who underwent IPC and thoracoscopic treatments. The identified priority of reducing time in hospital has been used to determine the primary outcome measure, with specific comments from patients suggesting that reducing time in hospital would ‘improve quality of life’. When asked about priorities in care, examples were (free text) ‘reducing time in hospital’, ‘to be able to breathe, to be as mobile as I can’ and ‘ease of breathing, support at home’. The study design has been discussed, and all surveyed would have been amenable to participation.

To ensure ongoing relevance of the study to patients, two PPI members sit on the TSC. All patient facing material has been reviewed by patients recruited from the North Bristol NHS Trust pleural clinic. For the purposes of the qualitative interview, a topic guide was developed in conjunction with the study’s PPI members and will be used to guide questions during the interview. This will be tested in two pilot interviews and revised following discussion with the participants and other members of the study team.

### Protocol changes following trial commencement

The trial details documented here are consistent with the TACTIC trial protocol V6.0 dated 14 December 2022. A summary of trial amendments can be found in the online supplemental appendix D.

## Ethics and dissemination

The study is sponsored in the UK by North Bristol NHS Trust and has been granted ethical approval by the London-Brent Research Ethics Committee (REC ref: 21/LO/0495).

TACTIC is registered on the ISRCTN public access database. Trial results will be presented at regional, national and international conferences with publication in a peer-reviewed journal (authorship will be listed according to journal guidelines). Raw data from the study will be made accessible to the public on request following completion and publication of the results.

## Discussion

The TACTIC trial is a multicentre RCT with the potential to radically affect how patients with malignant pleural disease are treated. It builds on work recently published within the field of pleural medicine. Both TTP and talc slurry via chest drain were shown to offer effective pleurodesis by Bhatnagar *et al*.^7^ The IPC-PLUS trial demonstrated that delivering talc slurry via an IPC did not lead to significant AEs such as drain blockage or septations, but offered more moderate pleurodesis success than hypothesised.^18^

TACTIC hypothesises that IPC insertion in the same procedure as TTP should combine the advantages of both approaches. A TTP will provide pleurodesis, immediately at time of thoracoscopy and allows for highly sensitive diagnostic biopsies to be taken in the same intervention. Addition of an IPC may allow the conversion of a TTP into a day case procedure and provide a ‘safety-net’ for ongoing fluid management in case of pleurodesis failure. Therefore, a combined procedure may provide more effective symptom control, better QoL and a reduction in healthcare utilisation.

While observational series have demonstrated encouraging findings from this approach, TACTIC will be the first trial to robustly challenge this hypothesis and importantly the first to assess outcomes prioritised by patients such as symptoms and QoL. By streamlining diagnostic and therapeutic interventions for MPE into a single ambulatory procedure, a positive trial outcome is likely to result in this approach being widely adopted by healthcare systems that are currently facing unprecedented strain.

## Trial status

Participant recruitment began in November 2021. There are currently 10 actively recruiting sites across the UK, with 54 participants enrolled (current to January 2023). Details of recruiting sites and principal investigators are included in online supplemental appendix E. Participant recruitment is expected to end in October 2023, with publication of results anticipated late 2024.

## Data Availability

Data sharing not applicable as no datasets generated and/or analysed for this study.
