# Supplementary material for: The randomised thoracoscopic talc poudrage+indwelling pleural catheters versus thoracoscopic talc poudrage only in malignant pleural effusion trial (TACTIC): study protocol for a randomised controlled trial
Source: BMJ Open Respir Res. 2023 May 30;10(1):e001682. doi: 10.1136/bmjresp-2023-001682 (PMC10230980; doi:10.1136/bmjresp-2023-001682)
Supplement: Supplementary data [file bmjresp-2023-001682supp001.pdf]

APPENDIX A: SCHEDULE OF STUDY PROCEDURES

**Trial visit / assessment**								
**Study Procedures**	Screening	Baseline assessment	Trial procedure (TTP or TTP+IPC)	Day 1 post procedure	Discharge	2 week FU	4 week FU	12 week FU
Time window		- 4 weeks		±24h		± 3 days	± 3 days	± 7 days
Eligibility assessment	x							
Informed consent		x						
VAS scores		x		x		x	x	
Randomisation			x					
Demographics / medical history / medications		x	x					
CXR		x	x			x	x	x
Thoracic US		x	x			x	x	x
Record of primary healthcare contact						x	x	x
Record of secondary healthcare contact						x	x	x
QOL assessments: EORTC QLQc30 and EQ5D5L		x					x	x
Care giver assessments *		x					x	x
Blood tests		x		x [a]				
Pleural fluid results		x						
Observations			x	x [a]	x			
Review record of daily IPC drainage vol						x	x	x
AE data collection			x	x	x	x	x	x
Semi-structured interview *								x

Footnotes:
* For those who consent
[a] N/a if discharged on day of procedure or if not taken as part of routine standard care

APPENDIX B: PATIENT DIARY EXTRACT

Trial number: TC ☐ - ☐☐☐ **Patients initials:** ☐☐

How to use this diary

We are collecting information about three important aspects of your care. In this diary, there is space to record:

1. Visual analogue scales (VAS) for symptoms of breathlessness and chest pain
2. Use of healthcare services after discharge from hospital
3. Volume of fluid drained at each IPC drainage (only applicable to patients receiving an IPC)

**Please bring this diary with you whenever you attend a trial-related appointment.**

Visual Analogue Scales (VAS)

Please fill in the charts about how much breathlessness and how much chest pain you are experiencing.

You should try to complete this at the same time of day each time where possible.

On each entry, please enter the date before placing a single vertical mark on the line to indicate the severity of your breathlessness (if any) and a single vertical mark on the line below to indicate the severity of your chest pain (if any).

An example is given below, but if you are still unsure, please ask a member of the trial team for help. Please do not write in the white area below the line.

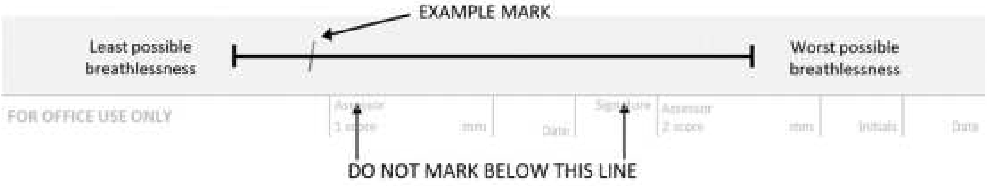

Please complete these charts twice per week, starting on the first day after your thoracoscopy procedure. The next VAS score will be recorded on day 5 after your procedure. After that, they can be completed twice per week on any days. They will be collected over a total of 4 weeks.

We can remind you to complete them with a weekly phone call. There is space on each page to record the dates as a reminder. A nurse from the trial team can help with this if needed.

TACTIC Participant Diary version 2.0 28Sept2021

Trial number: **TC** ☐ - ☐☐☐ **Patients initials:** ☐☐

WEEK ONE – VAS Scale

DAY 1 POST PROCEDURE

DATE: __ / __ / ____

Date and time of entry	DD	MM	YYYY		TIME	

HOW MUCH <u>BREATHLESSNESS</u> ARE YOU FEELING AT THE MOMENT?

No breathlessness at all |————————————————| Worst possible breathlessness

FOR OFFICE USE ONLY	Assessor	Initials	Date	Assessor	Initials	Date

HOW MUCH <u>CHEST PAIN</u> ARE YOU FEELING AT THE MOMENT?

No chest pain at all |————————————————| Worst possible chest pain

FOR OFFICE USE ONLY	Assessor	Initials	Date	Assessor	Initials	Date

DAY 5 POST PROCEDURE

DATE: __ / __ / ____

Date and time of entry	DD	MM	YYYY		TIME	

HOW MUCH <u>BREATHLESSNESS</u> ARE YOU FEELING AT THE MOMENT?

No breathlessness at all |————————————————| Worst possible breathlessness

FOR OFFICE USE ONLY	Assessor	Initials	Date	Assessor	Initials	Date

HOW MUCH <u>CHEST PAIN</u> ARE YOU FEELING AT THE MOMENT?

No chest pain at all |————————————————| Worst possible chest pain

FOR OFFICE USE ONLY	Assessor	Initials	Date	Assessor	Initials	Date

Trial number: TC ☐ - ☐☐☐ Patients initials: ☐☐

WEEK ONE - Healthcare needs diary

Please record any contact with healthcare providers you have had in the last week (for any reason).

	**No. times in the last week**	**Comments / reason for attendance** *E.g. I felt breathless*
Appointment with a general practitioner (GP) at the surgery		
Home visit from a GP		
Telephone / video consultation with a GP		
Hospital appointment with a doctor (face to face)		
Telephone / video consultation with a hospital doctor		
Treated in an accident & emergency (A&E) department		
I had to call for help from the ambulance services		
Admitted to hospital		
Appointment with a nurse at my GP surgery (face to face)		
Home visit from a nurse (do not include visit to drain IPC)		
Telephone / video consultation with a GP practice nurse		
Hospital appointment with a nurse (face to face)		
Telephone / video consultation with a hospital nurse		
Appointment with a physiotherapist		
Appointment with an occupational therapist		
Appointment with a psychologist		

WEEK ONE - Record of volume drained from IPC (Not applicable for patients who do not have an IPC (those randomised to 'standard care' group)).

Please ensure this record is completed EVERY time an attempt at draining the indwelling pleural catheter is made. If less than 50mls of fluid is drained on three consecutive occasions, please call local trial team.

	**DATE**			**VOLUME** (mls)	**COMMENTS** (Include reason for stopping)	**SIGN**
1	DD	MM	YYYY			
2	DD	MM	YYYY			
3	DD	MM	YYYY			
4	DD	MM	YYYY			
5	DD	MM	YYYY			
6	DD	MM	YYYY			
7	DD	MM	YYYY			

TACTIC Participant Diary version 2.0 28Sept2021

APPENDIX C: STATISTICAL ANALYSIS PLAN

1. Statistics and data analysis

1.1 Analysis Principals

The primary analysis for each outcome will be by intention-to-treat, meaning that all patients on whom an outcome is available will be included in the analysis, and will be analysed according to the treatment group to which they were randomised. All tests will be two-sided and will be considered statistically significant at the 5% level.

For each analysis, the following summaries will be provided:

a. The number of patients in each treatment group who are included in the analysis. The mean (SD) or median (IQR) in each treatment group for continuous outcomes, or the number and percentage of patients experiencing an event for categorical or time-to-event outcomes (time to-event outcomes will also present the median time to event in each treatment arm if applicable).

b The treatment effect (difference in means for continuous outcomes, standardized effect size, odds ratio for binary outcomes, hazard ratio for time-to-event outcomes, rate ratio for count outcomes) with its 95% confidence interval and a p-value.

The primary outcomes will be initially compared between groups (raw analysis) on an intention to treat basis. Adjusted analysis using regression for baseline imbalance and the minimisation factors will then be conducted to increase statistical power and certainty. Adjusted analyses for the minimisation variables will include the minimisation variables as covariates in the relevant regression model and will include i) type of underlying malignant disease [mesothelioma, other, unknown] and ii) WHO performance status [0-1 or 2-3]).

Missing data will be minimised but major remaining missing data (e.g. primary outcome) will be dealt with using sensitivity analyses.

1.2 Interim analysis

No Interim analyses are planned for this study. In the absence of a Data Monitoring Committee, the Oxford Respiratory Trials Unit (ORTU) Safety Oversight Group will advise the TSC on issues related to any safety data arising from the trial.

1.3 Sample size calculation

The study has two co-primary outcomes and separate sample size calculations are presented below. The null hypothesis states that there is no difference between the intervention (TTP+IPC) and standard care (TTP alone) in length of hospital stay over the first 4 weeks post randomisation, and in patient reported breathlessness over the first 4 weeks post randomisation.

1.3.1. Hospital stay:

Previous randomised studies demonstrate mean initial hospital stay in IPC treated patients of 0 days (SD 1) and in talc slurry pleurodesis of 4 days (SD 2). As talc poudrage pleurodesis is likely to result in shorter hospital stay than slurry, and this study captures hospital stay over 4 weeks rather than initial stay only, we have here conservatively assumed mean TTP+IPC stay over 4 weeks of 1 day, and mean TTP stay of 3 days, with a shared SD of 3 days to capture variability. Using these assumptions, **a total of 124** participants are required (two-sided alpha=5%, 95% power, including 5% attrition due to non-malignant diagnosis). This detected difference encompasses a clinically significant effect which would influence clinical decision making (i.e. reduction in hospital stay of 2 days total over one month, identified in patient surveys as a priority).

1.3.2. Breathlessness:

Previous randomised MPE studies demonstrate the mean breathlessness score in patients post treatment over 6 weeks is 25mm (SD 26mm) when treated with either talc or IPC. The MCID for VASd is 19mm. To detect a smaller difference than the MCID (to be conservative) of 16mm requires a total of 116 participants (two-sided alpha=5%, 90% power, including 5% attrition as above). Thus, the planned sample size of 124 participants is over-powered for this outcome.

1.4 Analysis of co-primary outcome variables

1. *Total number of days spent in hospital over 4 weeks post treatment.*
   (a) Unadjusted analyses will compare total number of days spent in hospital 4 weeks post treatment between randomised arms using the non-parametric log-rank test accounting for any censored data due to death defaulting to the Mann Whitney test in the absence of censoring. 95% confidence intervals for median differences will be reported.
   (b) Negative binomial regression will be used to compare randomised groups on this outcome including the minimisation factors as covariates.
2. *Average breathlessness over 4 weeks post treatment*
   (a) Unadjusted analyses will compare average breathlessness over 4 weeks post treatment between randomised arms using the non-parametric Hodges-Lehman test.
   (b) Linear regression (ANCOVA) will be used to compare randomised groups on this outcome including the minimisation factors as covariates.
   (c) Adjusted analyses will compare average breathlessness over four-week period between randomised arms using ANCOVA with baseline breathlessness and minimisation factors as covariates.

   Conclusions will be drawn from the adjusted analyses.

1.5 Analysis of secondary outcomes

1.5.1. Breathlessness Immediately Post Procedure.

(a) VAS breathlessness immediately post procedure will be compared between randomised arms using ANCOVA with baseline VAS breathlessness as a covariate.

1.5.2. *Time spent in hospital over 12-weeks.*

(a) Unadjusted analyses will compare total number of days spent in hospital over 12 weeks between randomised arms using the non-parametric log-rank test accounting for any censored data due to death defaulting to the Mann Whitney test in the absence of censoring. 95% confidence intervals for median differences will be reported.

(b) Negative binomial regression will be used to compare randomised groups on total number of days spent in hospital over 12 weeks including the minimisation factors as covariates.

1.5.3. *Time in hospital over 4 weeks accounting for participants who are suitable for discharge but remain in hospital for non-medical reasons.*

Time spent in hospital not counting time in hospital when otherwise suitable for discharge will be analysed.

(a) Unadjusted analyses will compare total number of days spent in hospital 4 weeks post treatment between randomised arms using the non-parametric log-rank test accounting for any censored data due to death defaulting to the Mann Whitney test in the absence of censoring. 95% confidence intervals for median differences will be reported.

(b) Negative binomial regression will be used to compare randomised groups on this outcome including the minimisation factors as covariates.

1.5.4. *Time in hospital over 12 weeks accounting for participants who are suitable for discharge but remain in hospital for non-medical reasons.*

Time spent in hospital not counting time in hospital when otherwise suitable for discharge will be analysed.

(a) Unadjusted analyses will compare total number of days spent in hospital 4 weeks post treatment between randomised arms using the non-parametric log-rank test accounting for any censored data due to death defaulting to the Mann Whitney test in the absence of censoring. 95% confidence intervals for median differences will be reported.

(b) Negative binomial regression will be used to compare randomised groups on this outcome including the minimisation factors as covariates.

1.5.5. *Average Chest Pain over four weeks post treatment.*

(a) Unadjusted analyses will compare average VAS Chest Pain over post 4 weeks post treatment between randomised arms using the non-parametric Mann Whitney test with 95% Hodges-Lehman confidence intervals.

(b) Adjusted analyses will compare VAS Chest Pain immediate post procedure between randomised arms using ANCOVA with baseline VAS Chest Pain as a covariate.

(c) Adjusted analyses will compare VAS Chest Pain immediate post procedure between randomised arms using ANCOVA with baseline VAS Chest Pain and minimisation factors as covariates.

1.5.6. *Chest Pain Immediately Post Procedure.*

(a) VAS Chest Pain immediate post procedure will be compared between randomised arms using ANCOVA with baseline VAS breathlessness as a covariate.

1.5.7. *Pleurodesis success at 4 and 12 weeks.*

(a) Unadjusted analyses will compare proportion and percentage of those achieving successful pleurodesis at 4-weeks between randomised arms using Fisher's exact test. 95% confidence intervals for the difference in success rates will be constructed using the Miettinen-Nurminen formula. 95% confidence interval for the proportion/percentage of successful pleurodesis at 4 weeks within each arm will be constructed using the Wilson score approach. The same analyses will be conducted for successful pleurodesis at 12-weeks.

(b) Adjusted analyses will compare proportion of those achieving successful pleurodesis at 4-weeks between randomised arms using binary logistic regression with minimisation variables as covariates. The odds ratio and 95% confidence interval will be reported. The same analysis will be conducted at 12-weeks.

1.5.8. *Number of healthcare contacts post discharge over 12 weeks.*

(a) Unadjusted analyses will compare number of primary care visits at 12-weeks post procedure between randomised arms using the non-parametric Hodges-Lehman test.

(b) Negative binomial regression will be used to compare randomised groups on this outcome including the minimisation factors as covariates.

1.5.9. *Quality of life at 4- and 12-weeks post procedure.*

EQ-5D-5L will be scored to give quality of life indices and EQ-5D-5L VAS rating. The EORTC QLQ-30 comprises 30 items, 24 of which are aggregated into nine multi-item scales, comprising five functioning scales (physical, role, cognitive, emotional and social), three symptom scales (fatigue, pain and nausea/vomiting) and one global health status scale. The remaining six single-item (dyspnoea, appetite loss, sleep disturbance, constipation, diarrhoea and the financial impact) scales assess symptoms. Scales and subscales are scored 0 – 100. All scales and subscales will be analysed in the same way at both 4- and 12-weeks.

(a) Unadjusted analyses will compare QoL measures between randomised arms using the non-parametric Hodges-Lehman test.

(b) Linear regression will be used to compare randomised groups on QoL measures including the baseline measure as a covariate.

(c) Linear regression will be used to compare randomised groups on QoL measures including the minimisation factors and baseline measure as covariates.

1.5.10. *Care-giver burden at 4- and 12-weeks post procedure.*

Burden is assessed using the SF-36 (8 domains, Physical Functions, Physical Limitations, Bodily Pain, General Health, Vitality, Social Functioning, Emotional problems, Mental Health each transformed between 0 - 100) and the General Health Questionnaire. All measures will be analysed in the same way at each of 4-weeks and 12-weeks.

(a) Unadjusted analyses will compare average burden at 4-weeks post treatment between randomised arms using the non-parametric Hodges-Lehman test. This analysis will be repeated at 12-weeks.

(b) Linear regression will be used to compare randomised groups on each measure of burden including the minimisation factors as covariates.

2. Subgroup analyses

Analyses will be conducted on the Intention-to-Treat analysis set. In addition, two pre-specified subgroups analyses will be undertaken. One subgroup will be all patients excluding those with trapped lung. A second subgroup will be all patients excluding those with a prolonged air leak after thoracoscopy - these patients could skew results. These subgroup analyses would be on the co-primary outcome measures. Any other subgroup analysis would be exploratory.

3. Sensitivity analyses

Primary analyses will be conducted using the Intention-to-Treat analysis set. The sensitivity of statistical conclusions with respect to the inclusion/exclusion of any clinically unusual, interesting, challenging or outlying cases will be undertaken including death. The robustness of statistical conclusions with respect to missing data will but undertaken on a missing-not-at-random (MNAR) basis using best-worst worst-best scenarios. If <5% of data is missing, then complete/available case analyses will be conducted. If 5% to 30% of data is missing, then best-worst worst-best sensitivity analyses will be undertaken. If more than 30% of data is missing, then results will be considered as hypothesis generating.

APPENDIX D: SUMMARY OF TRIAL AMENDMENTS

Amendment No.	Protocol version no.	Date issued	Author(s) of changes	Details of changes made
1	V4.0	10May2022	Emma Hedley	Page 4, change of email address for Emma Hedley and name change for Emma Keenan<br><br>Page 28, 7.5 additional information on patient with trapped lung<br><br>Page 29, 7.7 change of number of week VAS is recorded from 4 to 3<br><br>Page 29-30, 7.10 qualitative interviews, a change to not that patients can be interviewed without carers and addition of verbal consent.
2	V5.0	06Jul2022	Emma Hedley<br><br>Anand Sundaralingam	Page 5 – ORTU change of email address<br><br>Page 28 – removal of sentence regarding post procedure bloods as bloods are not required for the study.<br><br>Page 29 – Further clarifications within the last paragraph regarding approach for participant and carer consent for the qualitative interviews.<br><br>Page 49 – Footnote d – removal of the need for an additional CXR post chest tube removal as this is not standard care.
3	V6.0	14Dec2022	Alex Dipper<br>Emma Hedley	Page 12 – TMG personnel updates<br><br>Pages 34-36 updates to safety reporting. Removing IPC events from the anticipated list and simplifying the reporting process.

APPENDIX E: TACTIC RECRUITING SITES AND PRINCIPAL INVESTIGATORS

**Southmead Hospital, North Bristol**	Prof Nick Maskell (Chief Investigator)
**Churchill Hospital, Oxford**	Prof Najib Rahman
**Royal Preston Hospital, Preston**	Prof Mohammed Munavvar
**Derriford Hospital, Plymouth**	Dr John Corcoran
**Glenfield Hospital, Leicester**	Dr Rakesh Panchal
**Glan Clywd Hospital, Rhyl**	Dr Daniel Menzies
**Royal Stoke University Hospital, Stoke-on-Trent**	Dr Mohammed Haris
**King's Mill Hospital, Sutton-in-Ashfield**	Dr Mark Roberts
**Macclesfield District General Hospital, Macclesfield**	Dr Thapas Nagarajan
**Northern General Hospital, Sheffield**	Dr Duneesha De-Fonseka

Site set-up in progress:

**Wythenshawe Hospital, Manchester**	Dr Christopher Craig
